# Thrombin–Fibrin(ogen) Interactions, Host Defense and Risk of Thrombosis

**DOI:** 10.3390/ijms22052590

**Published:** 2021-03-04

**Authors:** Anne-Marije Hulshof, H. Coenraad Hemker, Henri M. H. Spronk, Yvonne M. C. Henskens, Hugo ten Cate

**Affiliations:** 1 Central Diagnostic Laboratory, Maastricht University Medical Centre, 6229 HX Maastricht, The Netherlands; yvonne.henskens@mumc.nl; 2 Department of Biochemistry, Cardiovascular Research Institute Maastricht (CARIM), Maastricht University, 6200 MD Maastricht, The Netherlands; henri.spronk@maastrichtuniversity.nl; 3 Synapse Research Institute, Cardiovascular Research Institute Maastricht, Maastricht University, 6200 MD Maastricht, The Netherlands; coen@hemker.nl; 4 Thrombosis Expert Centre Maastricht and Department of Internal Medicine, Section Vascular Medicine, Maastricht University Medical Centre, 6229 HX Maastricht, The Netherlands

**Keywords:** fibrinogen, thrombin, thrombin generation assay, immune system, inflammatory disease

## Abstract

Fibrinogen is a well-known risk factor for arterial and venous thrombosis. Its function is not restricted to clot formation, however, as it partakes in a complex interplay between thrombin, soluble plasma fibrinogen, and deposited fibrin matrices. Fibrinogen, like thrombin, participates predominantly in hemostasis to maintain vascular integrity, but executes some important pleiotropic effects: firstly, as observed in thrombin generation experiments, fibrin removes thrombin from free solution by adsorption. The adsorbed thrombin is protected from antithrombins, notably α2-macroglobulin, and remains physiologically active as it can activate factors V, VIII, and platelets. Secondly, immobilized fibrinogen or fibrin matrices activate monocytes/macrophages and neutrophils via Mac-1 interactions. Immobilized fibrin(ogen) thereby elicits a pro-inflammatory response with a reciprocal stimulating effect of the immune system on coagulation. In contrast, soluble fibrinogen prohibits recruitment of these immune cells. Thus, while fibrin matrices elicit a procoagulant response, both directly by protecting thrombin and indirectly through the immune system, high soluble fibrinogen levels might protect patients due to its immune diminutive function. The in vivo influence of the ‘protective’ plasma fibrinogen versus the ‘pro-thrombotic’ fibrin matrices on thrombosis should be explored in future research.

## 1. Introduction

The coagulation system, with fibrinogen to fibrin conversion as its final step, plays a crucial role in the restriction of bleeding and maintenance of vascular integrity. Fibrinogen is a 340-kDa plasma protein composed of Aα, Bβ and γ chains held together by disulfide and hydrogen bonds. The molecule has a trinodular structure with a single central E-domain and two outer D-domains, all linked by coiled-coil regions. Apart from albumin and the globulins, fibrinogen is the most abundant plasma protein and can be found in the human blood in physiological levels ranging from 2.0 to 4.0 g/L. Fibrinogen—also known as Factor I—primarily participates in the final step of coagulation as thrombin substrate and is converted to fibrin monomers by cleavage of fibrinopeptides A and B. Activated factor XIII (FXIIIa) then covalently connects multiple fibrin molecules into matrices to form a stable clot at the site of vascular damage. Additionally, fibrinogen is involved in the formation of platelet plugs. A variety of ligands (e.g., collagen and thrombin) can initiate stimulation of circulating platelets, which results in the conversion of αIIbβ3 from its resting low-affinity to its activated state [1]. This allows high-affinity binding of divalent fibrinogen, which bridges to activated αIIbβ3 molecules on adjacent platelets, provoking platelet aggregation [2]. Furthermore, the negatively charged phosphatidylserine (PS) becomes exposed in activated platelets, facilitating tenase and prothrombinase complex formation, thrombin activation, and fibrinogen conversion on the platelet surface [3]. The formed fibrin can bind GPVI and further activate platelets, resulting in more PS exposure. A positive feedback loop is established with fibrin mediated platelet activation further stimulating coagulation and thrombin activity [4].

High plasma fibrinogen levels can be provoked by an acute phase response in patients with tissue injury, pathogen invasion, and other inflammation inducing pathologies. These elevated fibrinogen levels are a result of inflammatory cytokines that stimulate the hepatic production of a variety of acute phase proteins. The increased hepatocyte production and excretion of fibrinogen is mostly mediated by interleukin (IL)-6, yielding massive increases in circulating plasma levels [5,6,7,8]. The dual function of fibrinogen as both coagulation factor and acute phase reactant raises the question whether or not an increase in fibrinogen level by itself induces the risk for thrombotic events. In the current review we evaluate thrombin–Fibrin interactions as observed in thrombin generation experiments, the importance of fibrinogen in host defense, and the thrombotic risk associated with high fibrinogen levels in inflammatory conditions and conclude that fibrinogen’s role within thrombosis is dependent upon its soluble or immobilized state.

## 2. Thrombin–Fibrin Interactions during Clot Formation

As early as 1945 it was observed that thrombin is adsorbed from free solution onto fibrin [9]. Fibrin therefore has been seen as an inhibitor of thrombin and was labeled ‘antithrombin 1′ [10]. This, however, is only one side of the medal as fibrin might as well be termed a thrombin protector.

The thrombin–Fibrin interactions described in the current paragraph are predominantly based on thrombin generation experiments as described by Hemker et al. [11]. In short, the thrombin concentration in clotting plasma is monitored by splitting a small fluorogenic substrate and comparing it to a constant known thrombin activity in a parallel, non-clotting sample. In clotting plasma there are two compartments, the clot and the surrounding fluid. If a fluorogenic substrate is added to the clotting plasma, the course of thrombin activity can be measured in both the clot and the surrounding fluid [12]. By subsampling from the surrounding fluid the thrombin activity in that compartment only will be measured [13,14]. Whether the subsampling technique is used or continuous thrombin activity in both compartments is measured has major implications for the interpretation of results, as will be discussed below. 

In both types of experiments a low molecular weight thrombin substrate is used to monitor thrombin activity. Such substrates are not only converted by free thrombin but also by thrombin bound to the inhibitor α2-macroglobulin (A2M). While antithrombin and other serine protease inhibitors (serpins) bind to the active site of thrombin and thus block any further activity, A2M is a large protein (800 kDa) that engulfs thrombin and thus prevents its action on any protein, including fibrinogen [15]. However, the thrombin active centre remains in a functional state, so that small molecular weight substrates can still be split. The measured thrombin activity in thrombin generation assays can thus be attributed to free and A2M-bound thrombin [16]. A mathematical procedure enables separate evaluations of the time course of free thrombin and the A2M-thrombin complex [16].

The role of fibrin can be reconnoitered by comparing the same plasma with and without fibrinogen. Fibrinogen can be removed from plasma without affecting the clotting system by producing a clot in citrated plasma through the addition of certain enzymes from snake venoms such as ancrod [17] or agihal [18] and winding out the clot. 

**Figure 1:**
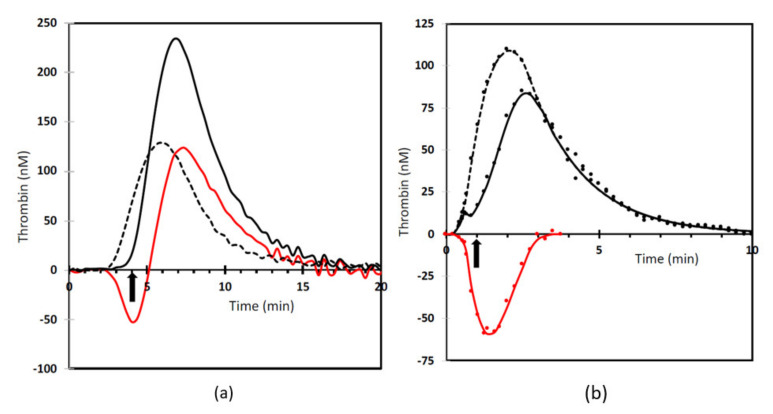
The effect of fibrin on thrombin generation. (**a**) measurement from added substrate where both the clot and fluid compartment are measured (based on [19]). (**b**) measurement using the subsampling method (based on [20]). Drawn line: normal plasma. Dotted line: defibrinated plasma. Red line: difference between normal and defibrinated plasma. The arrow indicates the moment of clotting.

In Figure 1a it is seen that in an experiment where both compartments are measured much more thrombin activity is measured in the presence of a clot than in its absence [19]. In contrast, when thrombin is measured in the fluid compartment only (Figure 1b), significantly less thrombin activity is achieved in the presence of a clot [20]. There must be a significant amount of thrombin that is adsorbed onto the clot and that is more resistant to inactivation than is thrombin in the free solution. This is further supported by the differences in thrombin decay between normal and defibrinated plasma. The overall thrombin decay is significantly slower in the presence of a fibrin clot, as presented in Table 1. Thrombin inactivation is due to complex formation with serpins and A2M. Both inhibitors perform better in defibrinated plasma as seen by the increase of A2M- and serpin-dependent decay constants in Table 1. In subsampling experiments (Figure 1b) it is seen that the downslopes in normal and defibrinated plasma coincide, suggesting that thrombin decay in the fluid compartment is not influenced by the presence of a fibrin clot. Therefore, the differences shown in Table 1 must be due to slow decay of thrombin in the clot department. This is most likely due to diffusion limitation of the interaction between inhibitors and fibrin-bound thrombin. The fact that the differences are much more outspoken for the large molecule A2M (800 kDa) than for the smaller serpins (e.g., antithrombin III; 58 kDa) favors this explanation.

There are several binding sites for thrombin on the fibrinogen molecule. The most prominent binding sites on fibrinogen are the sites where the fibrinopeptides split off, resulting in the formation of fibrin monomers. These vulnerable binding sites, ipso facto, disappear upon the formation of fibrin. Thrombin conversion of fibrinogen prevents the conversion of substrate in the thrombin generation assay. This explains the temporary dip in thrombin activity in the fluid phase at the moment of clotting (Figure 1b) and the lag in the appearance of measurable over-all thrombin activity (Figure 1a). On fibrin there remain low affinity reversible binding sites in the E-domain (K_a_ = 0.29 × 10^6^) and a high affinity binding site in the γ’ chain (K_a_ 4.9 × 10^6^) [21]. Thrombin has multiple binding sites for its ligands; exosite I, also called the fibrinogen-binding exosite, and exosite II, the main thrombin-inhibitor binding site [22]. Thrombin binds to γ’ fibrin via exosite II, making availability of the amidolytic center (exosite I) for further fibrinogen cleavage likely [23]. The γ’ chain is an alternatively spliced variant of the γ chain that occurs in approximately 15% of all fibrinogen molecules [24]. The more abundant γ chain is referred to as γA. Consequently, there are three forms of fibrinogen (and fibrin): γA-γA (85%), γA-γ’ (15%), and γ’-γ’ (<1%) [25]. 

That the protective effect of fibrin on thrombin is likely to be directly related to the γ-chain composition is demonstrated by the observation that a fibrinogen dependent increase in thrombin generation is smaller for γA-γA fibrin than for γA-γ’ fibrin, proportional to the concentration of the latter [26]. Additionally, the thrombin inhibitory effect of A2M during clotting is more pronounced in defibrinated plasma with added γA-γA fibrinogen compared to γA-γ’ fibrinogen [26]. This illustrates that in the presence of γ’ fibrin less free thrombin is available for inhibition by A2M. Thus, fibrin indeed protects thrombin from being inactivated and this effect is primarily due to the fact that A2M has limited access to thrombin molecules bound to γ’ chains.

Thrombin–Fibrin interactions do not only slow down thrombin decay by natural inhibitors, but also affect prothrombin conversion. The prothrombin conversion velocity curve can be calculated from the plasma thrombin decay constant and the thrombin generation curve [26]. It is then seen that prothrombin conversion in the absence of fibrin is lower (±15%) than in the presence of fibrin, independent of the fibrin type present. In accordance with this observation, serum prothrombin (after clot formation) was lower in the presence of fibrin than in its absence, 89 ± 11 nM vs. 172 ± 33 nM (mean ± SD), respectively [26]. This indicates more efficient prothrombin conversion in the presence of a fibrin clot. A tentative explanation might be that in the presence of a fibrin clot the pace of thrombin generation is not set by chemical processes but by diffusion [27]. Due to diffusion in the plane of the phospholipid surface on which the prothrombinase complex (and, by analogy, presumably the tenase complex) adsorbs, prothrombin conversion is the faster, the larger the phospholipid surface is [28]. It has been perceived that phospholipids of platelet microparticles spread on fibrin fibers [29]. Therefore, the presence of fibers may increase the available surface of procoagulant phospholipid and hence its efficiency to convert prothrombin.

It should be realized that in the presence of a fibrin clot, physical processes like flow and diffusion are at least as important as biochemical interactions between molecules., e.g., localization of active thrombin in a clot is partly the result of binding to fibrin but also occurs because of the sponge effect, i.e., because there is no flow within the clot. Taken together, the process of thrombin generation in the presence of a fibrin web may be completely different from that in solution.

The question remains of the biological function of clot bound thrombin. As previously mentioned, A2M inhibition of thrombin eliminates all biological functions in vivo but does allow conversion of the in vitro substrate. Fibrin bound thrombin does appear to retain its biological functions, however. The seminal experiments of Buchanan, that led to the discovery of thrombin (anno 1836) cannot be explained but by assuming that thrombin is adsorbed onto a clot, remained active overnight, could be extracted from the clot, and then induced clotting overnight [30]. Additionally, clots that are obtained by recalcification of plasma and thoroughly washed until the washing fluid was devoid of any detectable thrombin activity, shorten the factor Va-dependent lag-time of thrombin generation in the extrinsic system as well as that of the factor VIIIa-dependent thrombin generation in the intrinsic system [20]. Factor V or factor VIII preparations, that in themselves hardly influence thrombin generation patterns, acquire the capacity to shorten these lag-times when incubated with washed fibrin clots. Snake venom induced clots lacking fibrin are not active in this respect and neither is the last washing fluid of these plasma clots. Clots that are incubated in heparinized plasma are as active as clots from normal plasma are. It appears that clot-bound thrombin remains capable of activating factors V and VIII. A role of clot-bound factor Xa cannot be excluded but must be minor because a clot made by addition of thrombin to plasma from which the factors II, VII, IX and X have been removed is as active as a clot from normal plasma [20]. In addition to the activation of coagulation factors, it was shown that fibrin clots (irrespective of the presence of thrombin) provoked procoagulant activity in platelets. Although clot bound thrombin was not necessarily required for this phenomenon, thrombin significantly strengthens this increase [31].

In summary, fibrin does not function as an antithrombin, but removes thrombin from free solution by adsorption. The adsorbed thrombin is protected from antithrombins, notably A2M. It remains physiologically active as it can activate factors V, VIII, and platelets.

## 3. Fibrinogen and Host Defense

Fibrinogen can increase up to 10 g/L in severe inflammatory conditions. This increase is not necessarily associated with its hemostatic function, as physiologic fibrinogen concentrations (2.0–4.0 g/L) are sufficient for that purpose. However, the increase as an acute phase reactant may echo its immunological functions. The immune and hemostatic systems are closely intertwined [32], with the primary hemostatic defense function being the physical ‘capture’ and consequent sequestration of pathogens. Thrombin activity has been associated with improved survival after bacterial infection in several mice models [33,34,35]. Although part of this effect is due to platelet activation by thrombin via PAR1 (Protease Activated Receptor 1), thrombin-mediated fibrin deposition is the major protective mechanism [33]. Fibrinogen deficient (Fg^−/−^) mice show an increased bacterial burden, dissemination, and mortality as compared to wild-type or heterozygous (Fg^+/−^) mice [33,34,36,37]. Additionally, mice that carry normal levels of fibrinogen but generate less fibrin due to anticoagulant treatment (vitamin K antagonist; VKA or dabigatran) show increased infection related mortality [33,36,37]. Whether or not fibrinogen is beneficial after infection is both pathogen- and context-dependent.

The fibrin network plays a crucial part in the defense against pathogens. Mice with a mutant form of fibrinogen that lack the capacity to form fibrin polymers (Fib^AEK^), show decreased *Staphylococcus aureus* clearance and survival following intraperitoneal infection [38]. In other mice models, knockdown of the enzyme responsible for cross linking of polymerized fibrin, factor XIIIa, resulted in increased mortality and dissemination after bacterial infection [39,40]. These data suggest that fibrin polymerization is crucial for sequestration and elimination of pathogens. Nonetheless, the formation of fibrin matrices does not account for all fibrinogen mediated anti-microbial effects [38].

In addition to its hemostatic function, fibrinogen is an important regulator of inflammatory cell function and innate immunity [34,41]. Fibrinogen primarily interacts with the Mac-1 (CD11b/CD18, CR3, αMβ_2_) receptor expressed on neutrophils and monocytes/macrophages [42]. Mac-1 exhibits broad ligand recognition with over 40 different proteins capable of receptor binding and activation, including complement (C3bi), ICAM-1 and fibrin(ogen) [43,44]. Multiple binding sites in the fibrinogen γ-chain domains for Mac-1 have been described [44,45,46,47]. Notably, the Mac-1-fibrinogen affinity is dependent upon the fibrinogen condition; the binding motif is poorly exposed in soluble fibrinogen but becomes available for leukocyte interaction upon immobilization and in fibrin (both immobilized fibrinogen and fibrin are further referred to together as immobilized fibrin(ogen) in this paragraph) [48]. This mechanism allows soluble plasma fibrinogen to remain mostly invisible to circulating leukocytes, although, a low-affinity interaction with Mac-1 has been proposed. These differences between soluble fibrinogen and immobilized fibrin(ogen) already became apparent in 1990 as two research groups identified different Mac-1 interactions that they could not explain without presuming that distinct mechanisms were involved depending on the fibrinogen condition [49,50].

**Figure 2:**
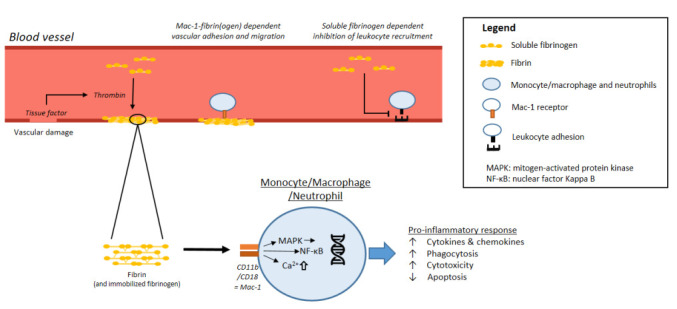
Fibrin(ogen)-immune system interactions.

In general, immobilized fibrin(ogen) induces a Mac-1 dependent pro-inflammatory response. [51] Immobilized fibrin(ogen)-Mac-1 interactions result in cell activation, illustrated by increased intracellular Ca^2+^, NF-ĸB and MAPK-pathway activation [41,52,53,54,55,56]. Additionally, both monocytes and neutrophils demonstrate an immobilized fibrin(ogen) dependent increase in adhesion, migration, phagocytosis, degranulation, cytokine/chemokine release, and apoptosis delay [35,52,55,56,57,58,59,60,61,62]. Soluble fibrinogen, however, is a relatively poor Mac-1 ligand and does not activate or induce signaling in neutrophils [48,63]. Nonetheless, circulating soluble fibrinogen levels partially diminish the inflammatory response. High concentrations of soluble fibrinogen, as present during acute phase reactions, dose-dependently reduce neutrophil adhesion under flow conditions [63,64]. These observations suggest opposite functions of soluble and immobilized fibrin(ogen), illustrated in Figure 2. Even though soluble fibrinogen is a poor ligand for Mac-1, the proposed low-affinity interaction between the two might execute its anti-inflammatory function; saturation of the Mac-1 receptor in milieus with high levels of soluble fibrinogen may compete with immobilized fibrin(ogen) for Mac-1 mediated leukocyte activation. Another hypothesis considers an auto-inhibitory effect of soluble fibrinogen through binding with its own immobilized or converted isoform. Soluble fibrinogen then forms a protective layer to shield the immobilized fibrin(ogen) and prohibit pro-inflammatory leukocyte adhesion and activation [65]. Taken together, soluble fibrinogen and immobilized fibrin(ogen) execute paradoxical effects with, respectively, an anti- and pro-inflammatory response.

The importance of fibrinogen in the host defense system is best illustrated by the number of bacterial factors that increase virulence by evasion from fibrin mediated capture. Several bacteria express fibrinolysis inducing proteins: *Group A streptococci* produce a streptokinase that mediates activation of the host fibrinolytic system through plasminogen to plasmin conversion [66,67]. This bacterial induction of fibrinolysis leads to further invasion from the primary infection site and dissemination to other organs [68]. Another example is the “Pla” surface protease produced by *Yersinia pestis* that induces clot breakdown and causes bacterial dissemination and thereby fulminant plague [69]. Thus, bacterial virulence increases when bacteria can invade and disseminate by escaping from the clot that arises from hemostatic activation at the site of infection.

In contrast, some bacteria use fibrinogen in their favor: *Staphylococcus aureus*, the most frequent cause of infective endocarditis, is characterized by the formation of thrombi composed of platelets, fibrin and bacteria [70,71]. *S. aureus* is known to hijack the host coagulation system to form a shield, protecting it from clearance by immune cells. Two well-described virulence factors are (staphylo)coagulase and Clumping Factor A (ClfA). Staphylocoagulase forms a 1:1 complex with prothrombin in which the active center, concealed in prothrombin, becomes exposed [72]; hence the complex is able to convert fibrinogen into fibrin. Additionally, staphylocoagulase reacts with des-gamma-carboxy prothrombin (protein induced by vitamin K absence II; PIVKA-II) [73]. PIVKA-II is produced in absence of vitamin K and does not contain the dicarboxyl group necessary to anchor itself via Ca^2+^ to the phospholipid layer. Therefore, thrombin is formed at a slower rate, which is desirable in patients receiving anticoagulants such as VKAs. However, staphylocoagulase converts PIVKA-II at the same rate as normal prothrombin [74]. Hence conventional anticoagulants cannot restrain coagulant activity in presence of staphylocoagulase. The complex between staphylocoagulase and prothrombin is capable of converting low molecular weight thrombin substrates [75]. Therefore, low molecular weight pseudo-substrates (i.e., Direct oral Anticoagulants; DOACs) that inhibit thrombin can block the active center of this complex and prevent further fibrinogen conversion. The ability of coagulases to induce prothrombin mediated fibrinogen conversion and to bind fibrinogen promotes bacterial shield formation [76]. Another virulence factor, fibrinogen γ-chain binding protein Clumping Factor A (ClfA), mediates bacterial adhesion to endothelial cells under shear conditions in a fibrinogen dependent manner [77,78]. Additionally, the formation of a fibrinogen “coat” impedes phagocytosis and can activate platelets by engaging the platelet integrin αIIbβ3, promoting thrombus formation [79,80,81]. Furthermore, as the name suggests, ClfA mediates bacterial ‘clumping’, which results in a phenotypic change from a primarily adhesive bacterium to a cytotoxic and invasive bacterium [82]. These fibrinogen-dependent characteristics promote bacterial dissemination, organ intrusion, and, consequently, virulence. Indeed, mice expressing mutant forms of fibrinogen, retaining clotting function but lacking the ClfA binding motif, exhibit reduced bacterial burden and organ damage [83]. When mice were infected with *Lactococcus lactis*, expressing mutant ClfA unable to bind fibrinogen, both dissemination and mortality were decreased when compared to mice infected with *Lactococcus lactis* bearing normal ClfA [84].

Another example includes *Group A streptococci* M1 protein, which binds fibrinogen. On the bacterial surface, fibrinogen binding to M1 prohibits phagocytic elimination of *Group A streptococci* by neutrophils as fibrinogen blocks deposition of antibodies and complement [85,86]. Additionally, free M1, released by neutrophil proteases, forms a cross-like pattern network with fibrinogen [87]. The M1-fibrinogen network is capable of activating neutrophils and triggers the release of heparin binding protein, promoting vascular permeability [88]. Fibrinogen favors *Group A streptococci* by forming a network that stimulates vascular leakage and prohibits phagocytic elimination. Thus, fibrinogen deficiency might prohibit bacterial dissemination and virulence after infection with pathogens expressing fibrinogen-binding proteins despite the previously described anti-microbial characteristics of this coagulation protein.

From a host perspective, fibrinogen mainly executes defensive functions, as coagulation end-product and by initiating a pro-inflammatory response. However, high levels of plasma fibrinogen might restrain this response by limiting leukocyte recruitment. Additionally, bacterial virulence factors can express proteins that mediate evasion of or profit from fibrinogen to stimulate dissemination and virulence. Thus, whether or not fibrinogen is beneficial after infection is both pathogen- and context-dependent. The acute phase reaction and consequent plasma fibrinogen increase might protect the host from collateral damage caused by the inflammatory response, contrary to locally immobilized fibrin(ogen) which marks the site of infection and/or vascular damage.

In physiological conditions, fibrin depositions will primarily develop in the extravascular matrix to recover vascular integrity, mark the affected site to leukocytes, and induce an appropriate inflammatory response as element of the wound healing process. However, excessive immobilized fibrin(ogen) deposition has been described in pathophysiological conditions. Fibrinogen is among the most common hemostatic factors associated with the development of atherosclerosis [89]. Atherosclerotic lesions, present in the intima of blood vessel walls and well-known precursors of cardiovascular thrombotic events, contain abundant fibrinogen [90]. Much of the fibrinogen in the plaque is cross-linked by tissue transglutaminase and accumulates as immobilized fibrinogen-matrices, suggested to be protected for thrombin conversion into fibrin [91]. This excessive insoluble fibrinogen accumulation might be involved in the low-grade inflammatory reaction fundamental to the atherosclerotic plaque and ensuing thrombosis. This is further supported by the notion that unstable plaques contain more fibrinogen (fragment D) than stable ones [92]. Additionally, disseminated intravascular coagulation (DIC) is characterized by elaborate thrombin activation resulting in intravascular clot formation and fibrin deposition [93]. Inflammation has a reciprocal effect on coagulation (reviewed in ref [94]), such as IL-6 dependent tissue factor expression. In DIC a vicious cycle is formed with inflammatory cytokines activating the coagulation system and, consequently, fibrin deposition further exacerbating the inflammatory response.

Besides functioning as a clotting factor, fibrinogen thus executes pleiotropic effects on the immune system: soluble fibrinogen inhibits leukocyte recruitment and immobilized fibrin(ogen) induces a pro-inflammatory response via Mac-1 interactions. High fibrinogen levels generally prohibit bacterial dissemination from the site of infection. However, several bacteria express factors that mediate its virulence by means of fibrinogen interaction. Although context dependent, fibrinogen interactions with the immune system are important for host defense.

## 4. Fibrinogen Level and Thrombotic Risk in Inflammatory Diseases

In the early 80s the positive association between plasma fibrinogen level and long-term risk of cardiovascular disease was first described [95,96]. Nowadays, it has been generally accepted that fibrinogen is an independent risk predictor of cardiovascular events [97]. A role for fibrinogen as risk factor in the development of deep venous thrombi has also been reported [98]. Even though the increased thrombotic risks of elevated fibrinogen levels are well established, a clear causative (genetic) connection between fibrinogen level and thrombotic risk has not yet been found [99,100,101].

Inflammatory conditions cause hemostatic alterations and are also known to be predictive of thrombosis. These changes precipitate a prothrombotic tendency and consist of an increase in procoagulant factors, inhibition of natural anticoagulants, and diminished fibrinolysis [102]. Thus, the rise in fibrinogen is a single entity in a complex interplay of coagulation and inflammation.

An important distinction should be made between acute and chronic low-grade inflammation. Chronic inflammation is characterized by mild elevations in hsCRP (high sensitivity C-Reactive Protein), fibrinogen, and other inflammatory markers. People with diseases characterized by chronic low-grade inflammation, such as diabetes and metabolic syndrome, carry higher fibrinogen levels and are at an increased risk for cardiovascular disease [103,104,105,106]. Acute inflammatory conditions are distinctively different: within a short period of time inflammation markers show substantial elevation as a component of the acute phase reaction. As described previously, fibrinogen is an acute phase protein and massively increases during such pathologies. Patients with sepsis, one of the most pronounced acute inflammatory conditions, typically present with systemic microthrombosis and an increased risk for venous thromboembolisms [107,108]. Though having substantially different underlying pathologies, both acute and chronic inflammatory conditions carry an increased thrombotic risk, have elevated fibrinogen levels, and an activated immune system.

Notably, total plasma fibrinogen is not only elevated in inflammatory conditions, the fibrinogen composition is altered as well. The alternatively spliced γ’ fibrinogen becomes elevated in chronic and acute inflammation, both in absolute amounts and as a ratio to total fibrinogen [109,110,111]. As previously described, the γ’ chain contains a high affinity thrombin binding site. In line with the thrombin generation observations, it would be expected that an increase in γ’ fibrinogen is associated with more thrombin generation and possibly thrombosis. Fibrin clots produced from purified γA-γ’ show a structure that has previously been related to thrombotic risk: reduced average fiber diameter, increased branching, and reduced pore size [112]. Several cardiovascular studies have shown an association between elevated γ’ fibrinogen level and cardiovascular events which was independent of the rise in total plasma fibrinogen in some studies [113,114,115,116]. It should be noted that prospective studies currently lack, making it difficult to establish whether the elevation in γ’ fibrinogen can be seen as a cause or consequence. In a carotid arterial thrombosis mouse model, injection with γA-γA rather than γA-γ’ resulted in more thrombosis [117]. Additionally, when correcting for CRP, the primary inflammatory marker, the association between γ’ fibrinogen and cardiovascular disease was eliminated. These results suggest γ’ fibrinogen to be a general marker of inflammation, rather than an independent risk factor for cardiovascular disease [115].

In contrast to arterial thrombosis, lower γ’ fibrinogen and γ’/total fibrinogen ratios in patients were associated with an increased risk for venous thrombosis and microangiopathy, suggesting a protective effect of γ’ chains [118,119]. Additionally, in a prothrombotic factor V Leiden venous thrombosis mouse model, a moderate protective effect after γA-γ’ fibrinogen injection was seen when compared to γA-γA fibrinogen addition [120]. However, this study was unable to reproduce a similar effect in wild type mice.

Taken together, the exact thrombotic risk associated with the elevation of γ’ fibrinogen in inflammatory conditions is unclear at the moment. In vitro studies demonstrated that thrombin bound to the high affinity γ’ chain binding site had diminished platelet activation capacity and was unable to bind fibrinogen [121,122]. This however does not necessarily prove that increased γ’ results in less thrombin activity in vivo. In the circulation γ’ fibrinogen may function as thrombin depository, where it remains protected from inhibitory molecules and diffusion but becomes available for substrate due to the reversible binding site [121].

Both acute and chronic inflammation are characterized by increased total plasma fibrinogen and the ratio of γ’ fibrinogen, though to a different extent. Thrombin generation experiments with γ’ fibrinogen suggest an increased thrombotic risk at higher concentrations of this alternatively spliced fibrinogen variant. However, the effect of γ’ fibrinogen on both arterial and venous thrombotic risk remains inconclusive.

## 5. Synthesis: Balancing Inflammation and Coagulation

Thrombosis is without exception preceded by hemostasis and the formation of fibrin matrices. Additionally, epidemiologic studies consistently associated plasma fibrinogen level with thrombotic risk, in particular atherothrombosis. Not surprisingly, it is presumed sensible to consider all elevated fibrinogen entities as prothrombotic markers. However, the rise of plasma fibrinogen as an acute phase reactant in inflammatory conditions might prevent inflammatory disarray and thrombotic complications by its immune diminutive properties. One could hypothesize that high plasma fibrinogen in itself does not cause thrombosis. Fibrin matrices, on the other hand, might induce a vicious cycle that, in some instances, results in thrombus formation. Excessive deposition of fibrin might induce thrombosis via two positive feedback mechanisms: (1) the thrombin protective characteristics of fibrin matrices further induce coagulation, (2) the pro-inflammatory response provoked by fibrin deposition has a reciprocal, stimulating effect on coagulation. This scenario presumes that individuals with high plasma fibrinogen require a second hit to induce hemostasis and convert the ‘protective’ soluble fibrinogen into the ‘pro-thrombotic’ fibrin matrices. Unquestionably, an individual does not develop thrombosis at every instance vascular repair by hemostasis is required. Thrombosis is generally preceded by a multitude of signals and complex hemostatic changes eventually resulting in an inevitable disbalance towards a prothrombotic phenotype. Excessive fibrin deposition thus might initiate or aggravate this disbalance due to its thrombin protecting and immune stimulating properties.

This scenario has become highly relevant in the recent COVID-19 pandemic, where patients infected with SARS-CoV-2 suffer from a coagulopathy that, depending on disease severity, shows profound elevations in fibrinogen levels, and also a high rate of pulmonary thrombosis. Based on the current dogma, the observed high fibrinogen level in COVID-19 patients could be linked to the frequent occurrence of thrombosis. On the other hand, Thachil suggested that high fibrinogen does not necessarily cause pulmonary thrombosis, but rather protects against it, due to its pleiotropic effect on the immune system [123]. Indeed, in a longitudinal analysis of severely ill COVID-19 patients, low rather than high fibrinogen was associated with pulmonary embolism [124]. This is in line with the concept that high plasma fibrinogen levels elicit a protective function. It should be noted, that ex vivo assays can only assess the in vivo plasma fibrinogen level. Therefore, a possible discrepancy exists between the measurable plasma fibrinogen level and the total amount of both plasma and immobilized fibrin(ogen). Several scenarios may present in a patient. One scenario considers DIC associated depletion of plasma fibrinogen; fibrinogen measurements might be low, but abundant microthrombi suggest a large amount of fibrin deposition. Another scenario includes the pregnancy related increase in plasma fibrinogen level, which does not abruptly result in major fibrin deposition. In COVID-19 patients the increase in several markers, including D-dimer and prothrombin 1&2, support the presence of fibrin deposition [125]; in combination with high plasma fibrinogen levels this illustrates a third scenario in which both the soluble and immobilized fibrinogen compartments are enhanced. Taken together, plasma fibrinogen levels as measured by ex vivo assays are unlikely to reliably reflect the immobilized compartment and total fibrinogen. The discrepancy between measurable soluble fibrinogen and unmeasurable immobilized fibrin(ogen) complicates analyzing the individual effect of these distinct fibrinogen entities.

Fibrinogen, like thrombin, participates not only in hemostasis but executes multiple pleiotropic effects including immune modulation. High plasma fibrinogen might protect patients during acute inflammatory pathologies due to its immune diminutive function. Rather than simply being the plug that is the upshot of hemostasis and thrombosis, fibrin matrices elicit a procoagulant response, both directly by protecting thrombin as it incorporates into the clot, but also indirectly through the immune system. Fibrinogen is involved in a complex balancing act between thrombin, soluble plasma fibrinogen, and deposited fibrin matrices. Future research should focus on the importance of different fibrinogen entities on the hemostatic balance preceding thrombosis. More specific biomarkers identifying both the amount of soluble and immobilized fibrin(ogen) could result in better thrombosis risk prediction for patients.

## Figures and Tables

**Table 1:** Decay constants of thrombin in normal and defibrinated plasma using thrombin generation with an added substrate where both the clot and fluid compartment are measured. From [26].

	Normal Plasma	Defibrinated Plasma
Total	0.40	0.70
α2-macroglobulin	0.05	0.18
Serpins	0.35	0.47

Presented are first order decay constants expressed in min^−1^.
